# Sugar binding of sodium–glucose cotransporters analyzed by voltage-clamp fluorometry

**DOI:** 10.1016/j.jbc.2024.107215

**Published:** 2024-03-24

**Authors:** Erika Watabe, Akira Kawanabe, Kazuyo Kamitori, Satoko Ichihara, Yuichiro Fujiwara

**Affiliations:** 1 Laboratory of Molecular Physiology & Biophysics, Faculty of Medicine, Kagawa University, Miki-cho, Kagawa, Japan; 2 International Institute of Rare Sugar Research and Education, Kagawa University, Miki-cho, Kagawa, Japan; 3 Laboratory of Physiology and Biophysics, Graduate School of Biomedical and Health Sciences, Hiroshima University, Hiroshima City, Hiroshima, Japan

**Keywords:** membrane transport, sugar transport, structure-function, substrate specificity, voltage-clamp fluorometry, SGLT, substrate recognition

## Abstract

Sugar absorption is crucial for life and relies on glucose transporters, including sodium–glucose cotransporters (SGLTs). Although the structure of SGLTs has been resolved, the substrate selectivity of SGLTs across diverse isoforms has not been determined owing to the complex substrate-recognition processes and limited analysis methods. Therefore, this study used voltage-clamp fluorometry (VCF) to explore the substrate-binding affinities of human SGLT1 in *Xenopus* oocytes. VCF analysis revealed high-affinity binding of D-glucose and D-galactose, which are known transported substrates. D-fructose, which is not a transported substrate, also bound to SGLT1, suggesting potential recognition despite the lack of transport activity. VCF analysis using the T287N mutant of the substrate-binding pocket, which has reduced D-glucose transport capacity, showed that its D-galactose-binding affinity exceeded its D-glucose-binding affinity. This suggests that the change in the VCF signal was due to substrate binding to the binding pocket. Both D-fructose and L-sorbose showed similar binding affinities, indicating that SGLT1 preferentially binds to pyranose-form sugars, including D-fructopyranose. Electrophysiological analysis confirmed that D-fructose binding did not affect the SGLT1 transport function. The significance of the VCF assay lies in its ability to measure sugar–protein interactions in living cells, thereby bridging the gap between structural analyses and functional characterizations of sugar transporters. Our findings also provide insights into SGLT substrate selectivity and the potential for developing medicines with reduced side effects by targeting non-glucose sugars with low bioreactivity.

Sugar absorption is one of the most important functions of life. Ingested carbohydrates are digested into monosaccharides, such as glucose and fructose, which are absorbed into the blood by the epithelial cells of the small intestine (1). Glucose transporters play a role in the cellular absorption of monosaccharides. There are two types of glucose transporters: sodium–glucose cotransporters (SGLTs), which exist in the epithelial cell membranes of the intestine and kidneys, and glucose transporters, which exist widely in the membranes of tissues, such as the pancreas, muscle cells, and blood–brain barrier (2, 3). Each protein has several isoforms with different expression patterns, substrate specificities, and transport dynamics (4).

SGLTs are transporters of the SLC5 gene family that cotransport sodium ions and monosaccharides into cells (3). SGLT1 is mainly found in the small intestine and transports D-glucose and D-galactose, and mutations in this transporter cause malabsorption (5, 6). SGLT2 in kidney tubules reabsorbs D-glucose, a key target in diabetes treatment (7, 8, 9). SGLT3 acts as a glucose sensor (10), SGLT4 and SGLT5 support D-fructose and D-mannose absorption (11), and SGLT6, also known as SMIT, transports myoinositol (12). Different SGLTs exhibit specific sugar selectivities and physiological effects (3). Moreover, monosaccharide substrate structures vary, with D-fructose and D-glucose exhibiting different structures, such as furanose and pyranose structures, respectively. Even for sugars with the same pyranose structure as glucose, the orientation of hydroxyl groups on the pyranose carbon skeleton may differ from that of D-glucose, thus defining a variety of sugars (*e.g*., galactose and mannose) (13). SGLT recognizes these structural differences and selects substrates accordingly.

To date, substrate transport by SGLT *in vivo* has been analyzed using knockout mouse experiments (14), RNA interference techniques (15), and human pathogenic mutation analyses (5). *In vitro* identification of transported substrates has been performed by analyzing the uptake of radioisotope-labeled (RI) substrates (16, 17), performing electrophysiological analyses using cotransported sodium as a charge carrier (18, 19, 20), and conducting liposome reconstitution system assays (21). Advances in structural biology have provided clues to explain the substrate specificity of glucose transporters. The structure of SGLT was initially determined by analyzing the sodium/galactose symporter of *Vibrio parahaemolyticus* (22), and the structures of human SGLT1 and SGLT2 have recently been assessed (23, 24). Although substrate specificity or transport processes have been discussed based on the structure of the substrate, few reports have explained the process from structure to function. Thus, the mechanisms underlying the recognition of various substrates by interactions with binding pockets have not been clarified. Although studies have analyzed the affinity for transported substrates or derivatives using binding pocket mutants, certain experimental data cannot be directly explained by the structure of the binding pocket; nonetheless, substrate selectivity may be acquired through binding sites other than the known binding pocket or through changes in the binding structure during transport (18, 20, 25). Most methods for analyzing transporter function analyze transported substrates and cannot separate substrate binding steps from substrate transport steps. Thus, they provide insufficient evidence to explain substrate specificity, and methodological breakthroughs are required.

Voltage-clamp fluorometry (VCF) analysis, in which fluorescent labels are added to membrane proteins and structural changes are analyzed based on their fluorescence, has achieved great success in structure-function relationship studies of ion channels and membrane enzymes (26, 27). In recent years, the VCF method has been modified to study transporters and applied to estimate conformational changes during substrate transport (28, 29, 30). In this study, we used the VCF method to analyze the binding steps between cell membrane-expressed SGLT molecules and substrates containing various sugars.

## Results

**Figure 1:**
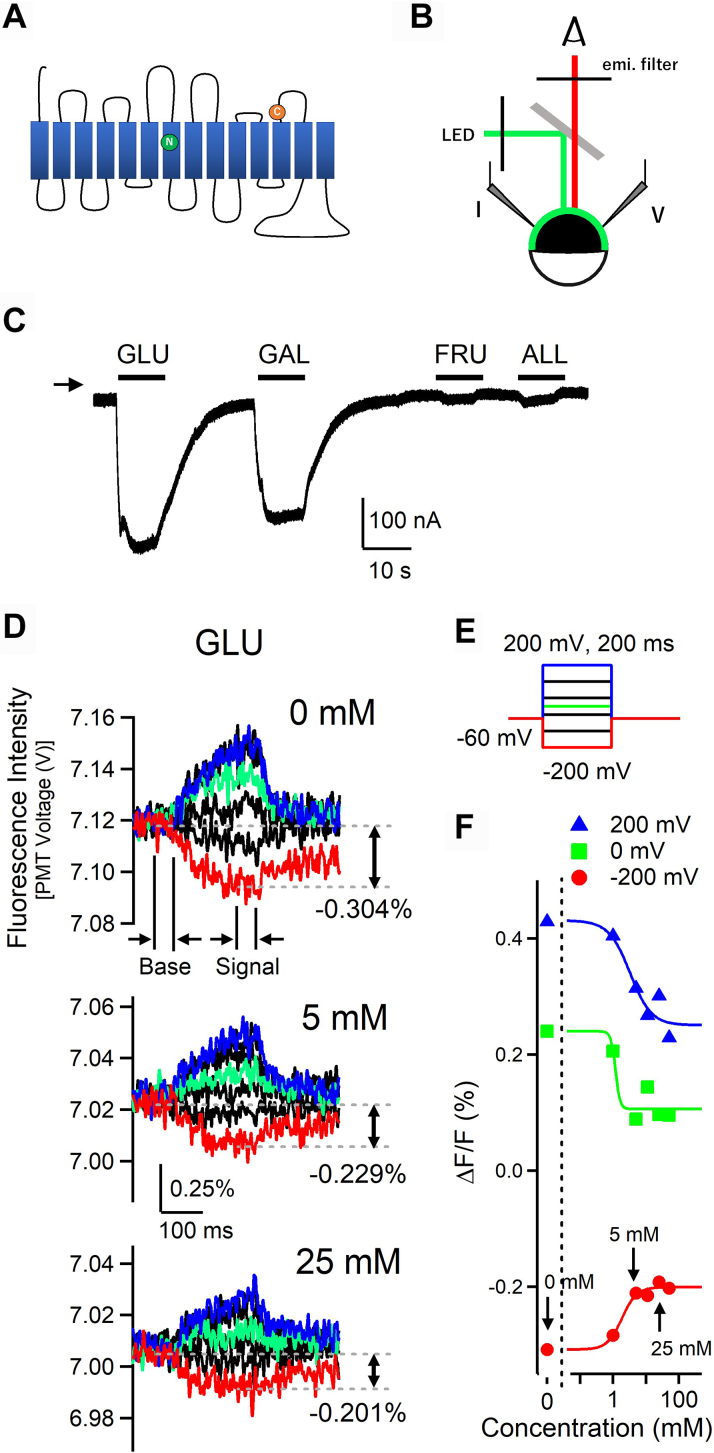
**Effects of D-glucose on the voltage-induced fluorescence changes of hSGLT1.***A*, schematic drawing of hSGLT1 construction for voltage-clamp fluorometry (VCF). Cysteine was introduced into the extracellular Gly-507 (*orange*) of the 12th transmembrane segment, and MTS-TAMRA was bound. Asparagine was introduced at Thr-287 (*green*) in the seventh transmembrane segment in the experiment in Figure 4. *B*, machine setup for VCF. Excitation light and fluorescence were bifurcated by a dichroic mirror. *C*, representative current trace of hSGLT1 G507 by administration of 10 mM sugars (*black bars*). An *arrowhead* indicates the zero level of the current. *D*, representative voltage-induced fluorescent traces of hSGLT1 measured in the presence of different concentrations (0, 5, and 25 mM) of D-glucose. The traces recorded at various membrane potentials shown in (*E*) are overlaid. *Red*, *green*, and *blue* traces indicate the fluorescence signal obtained by −200 mV, 0 mV, and +200 mV voltage step pulses, respectively. Changes in signal fluorescence value from the base fluorescence value were analyzed. *E*, pulse protocol for VCF. Membrane potential was held at −60 mV and determined at 200 ms step pulses of −200, −120 mV, −40 mV, 0 mV, +40 mV, +120 mV, and +200 mV. *F*, dose–response relationships of the normalized fluorescent intensity of hSGLT1 with D-glucose. Normalized fluorescence intensity (*ΔF/F*) was obtained by dividing the change in fluorescence under test pulses (*ΔF*) by the baseline fluorescence intensity at the holding potential (F), where the average values of the “baseline” and “signal” regions in (*D*) were used. Data were analyzed for −200 mV, 0 mV, and +200 mV. *ΔF/F* values at the end point region of test pulse in (*D*) were plotted (*red*, *green*, and *blue circles*) and fitted by the Hill equation. Data for 0, 1, 5, 11, 25, and 50 mM were plotted. Apparent Kd values were 1.8 mM for −200 mV, 1.2 mM for 0 mV, and 3.3 mM for +200 mV. ALL, D-allose; FRU, D-fructose; GAL, D-galactose; GLU, D-glucose; MTS-TAMRA, 2-((5(6)-tetramethyl-rhodamine)carboxylamino)ethyl methanethiosulfonate; SGLT, sodium–glucose cotransporter.

**Figure 2:**
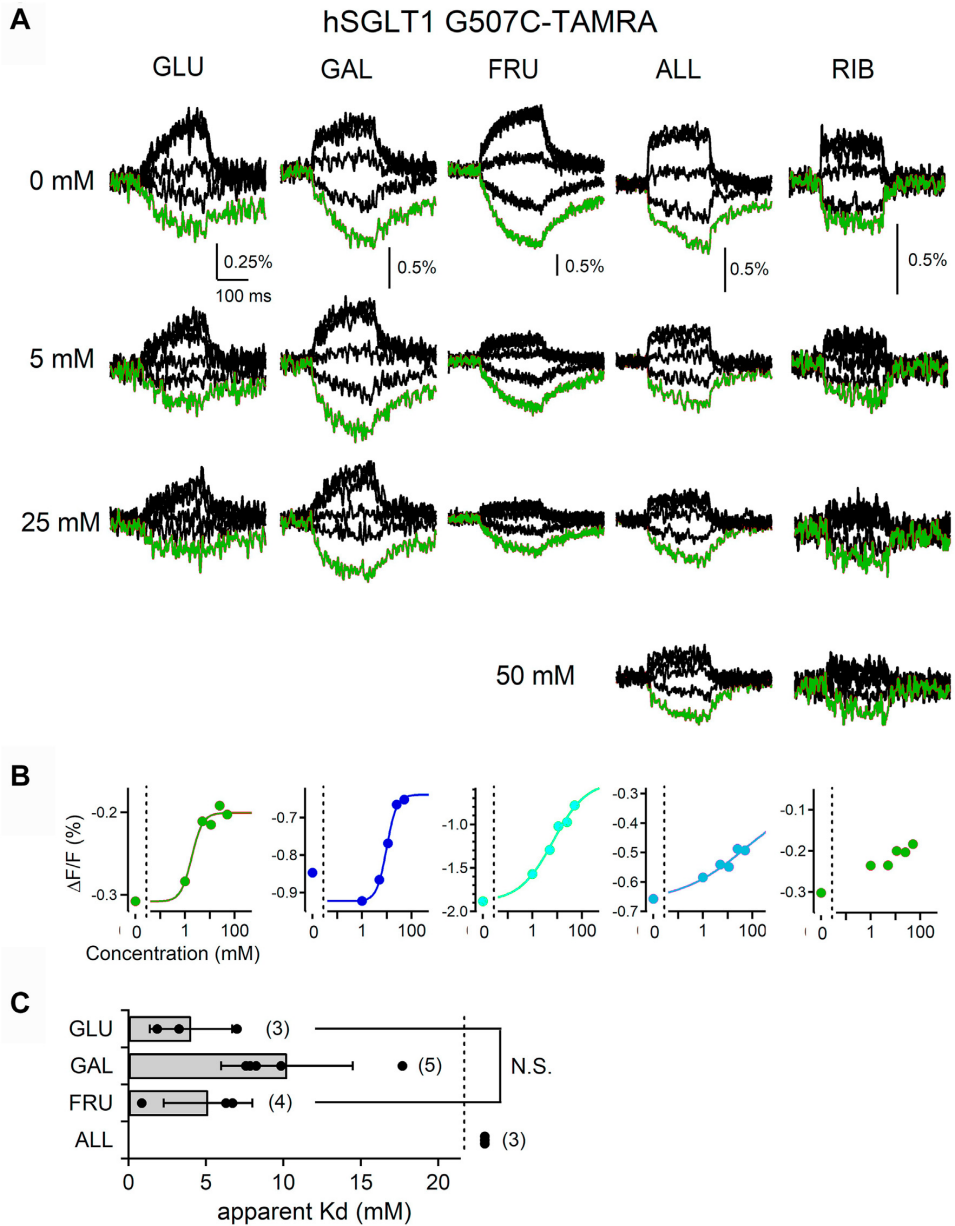
**Effects of sugars on the voltage-induced fluorescence changes of hSGLT1.***A*, representative voltage-induced fluorescent traces of hSGLT1 measured at different concentrations of D-glucose, D-galactose, D-fructose, D-allose, and D-ribose. Step pulses were applied as in Figure 1*D*. The red trace shows the fluorescence signal obtained by a −200 mV voltage step pulse. Fluorescence traces of D-glucose from Figure 1*D* were used for comparison. *B*, dose–response relationships of the normalized fluorescent intensity of hSGLT with D-glucose (*red*), D-galactose (*blue*), D-fructose (*green*), D-allose (*pink*), and D-ribose (*black*). Fluorescent values at the end point of the test pulse at the −200 mV step in (*A*) were plotted (*colored circles*). Hill-fitted curves are also shown. The apparent Kd value for D-allose was too high and thus was not reliable, and D-ribose could not even be fitted. Data were collected from the same oocytes described in (*A*), and data for 1, 5, 11, 25, and 50 mM were plotted. *C*, comparison of the apparent Kd values. Bars indicate means ± standard deviation (SD), and all data are plotted as *black dots*. Numbers in brackets indicate the number of experiments (n = 3–5). Data were statistically analyzed *via* one-way ANOVA with Tukey–Kramer’s post hoc test (N.S., *p* > 0.05). ALL, D-allose; FRU, D-fructose; GAL, D-galactose; GLU, D-glucose; RIB, D-ribose; SGLT, sodium–glucose cotransporter.

**Figure 3:**
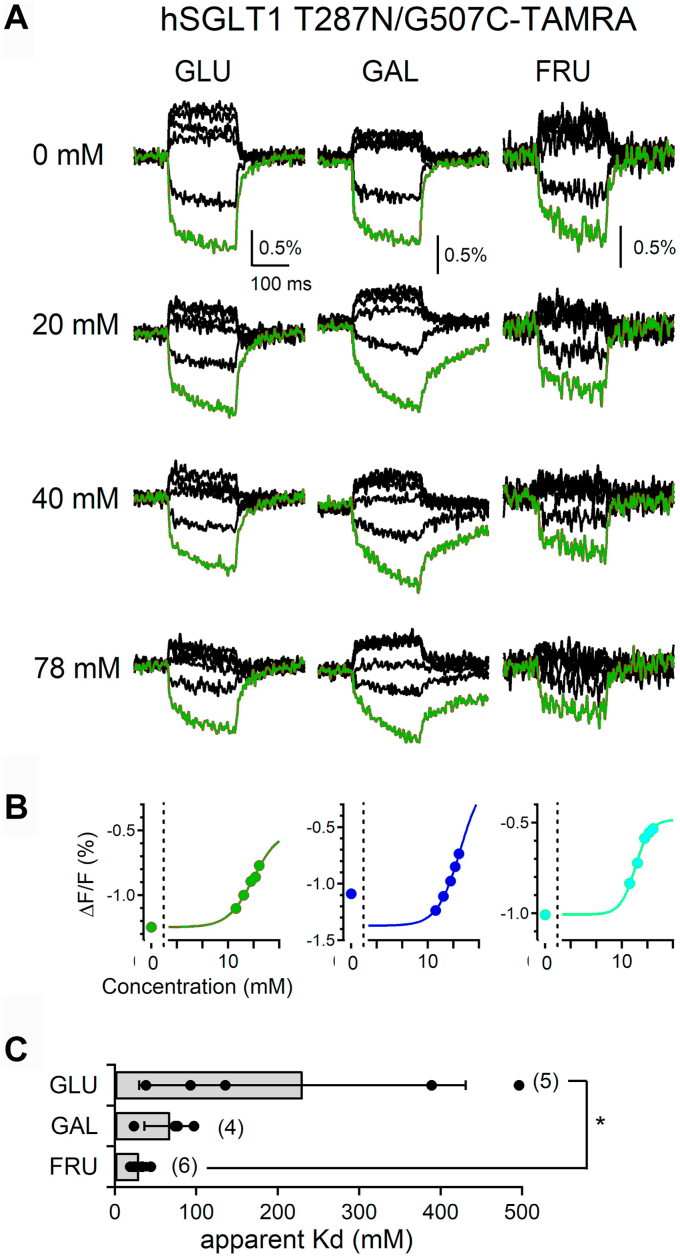
**Effects of sugars on the voltage-induced fluorescence changes of hSGLT1 T287N.***A*, representative voltage-induced fluorescent traces of the hSGLT1 T287N mutant with different concentrations (0, 20, 40, and 78 mM) of D-glucose, D-galactose, and D-fructose. Step pulses were applied as in Figure 1*D*. *Red traces* show each fluorescent signal obtained by the −200 mV step pulse. *B*, dose–response relationships of the fluorescent intensity of T287N with D-glucose (*red*), D-galactose (*blue*), and D-fructose (*green*). Fluorescent values at the end point of the test pulse at the −200 mV step in A were plotted for 20, 40, 78, 116, and 165 mM (*colored circles*), and Hill-fitted curves are also shown. *C*, comparison of the apparent Kd values. Bars indicate the means ± SD, and all data are plotted as *black dots*. Numbers in brackets indicate the number of experiments (n = 3–6). Data were statistically analyzed *via* one-way ANOVA and Tukey–Kramer’s post hoc test (∗*p* = 0.037 < 0.05). FRU, D-fructose; GAL, D-galactose; GLU, D-glucose; SGLT, sodium–glucose cotransporter.

Each member of the SGLT family contains 14 transmembrane domain proteins (Fig. 1*A*), and structural biological analysis revealed the approximate locations of the binding domains for glucose and Na^+^ within the transmembrane region. In this study, we investigated the substrate selectivity of human SGLT1 that is responsible for D-glucose and D-galactose transport. To examine the interactions between SGLT and its substrates, we utilized the VCF technique by adapting methods described in previous studies. Moreover, 2-((5(6)-tetramethyl-rhodamine)carboxylamino)ethyl methanethiosulfonate (MTS-TAMRA) was added to human SGLT1 expressed in *Xenopus* oocytes, and the fluorescent signal emitted by MTS-TAMRA was analyzed using LED excitation light (Fig. 1, *A* and *B*). Because MTS-TAMRA changes its fluorescence signal intensity depending on the hydrophobic environment in its vicinity, MTS-TAMRA was attached to Cys (G507C), which was mutated to G507. This mutation is located at the extracellular interface of SGLT molecules, far from the substrate-binding pocket (Fig. 1*A*). The G507C construct was analyzed for sugar transport currents using the two-electrode voltage-clamp method. Currents induced by 10 mM glucose and galactose were observed, while few currents induced by allose and fructose were observed (Fig. 1*C*). These results were consistent with wildtype hSGLT1; thus, the G507 construct was used for subsequent VCF analysis. Representative fluorescence signal traces before and after D-glucose administration are shown in Figure 1*D*, and the applied step pulses are presented in Figure 1*E*. The fluorescence signal was attenuated over time by the application of hyperpolarizing voltage and increased over time by the application of depolarizing voltage. Normalized VCF signal traces based on the VCF signal at a holding potential of −60 mV are presented in Figure 1*D*. The change in fluorescence signal intensity at each voltage (*ΔF*) was divided by the baseline fluorescence intensity at the holding potential (*F*) to obtain the normalized fluorescence intensity (*ΔF/F*). The fluorescence intensity values were averaged over 50 ms to avoid noise effects in the analysis (Fig. 1*D*). The noise level of the VCF signal was comparable to that reported for other ion channels and membrane enzymes (31, 32, 33). In the absence of D-glucose, the SGLT1 G507C-TAMRA oocytes exhibited a change in fluorescent signal under a voltage step pulse, with the largest amplitude of the fluorescent signal observed at a hyperpolarization of −200 mV (Fig. 1*D*). When D-glucose was administered to the oocytes, the amplitude of the fluorescent signal decreased as the concentration of D-glucose increased (Fig. 1*D*). This change in fluorescence emitted from MTS-TAMRA was attributed to the conformational change in SGLT before and after substrate binding; therefore, the apparent sugar affinity of SGLT can be determined by analyzing the fluorescence changes. The relationship between the concentration of sugar and the change in fluorescence intensity measured when −200 mV, 0 mV, and +200 mV pulses were applied (dose-*ΔF/F* relationship) is shown in Figure 1*F*. The apparent affinity of glucose for hSGLT1 did not show voltage dependence. Similar to the analysis of other sugars, the data obtained at -200 mV produced a stable maximum VCF signal that was used in the following analysis and discussion (Figs. 1, 2, 3, and 5). The apparent dissociation constant (Kd) value at −200 mV was 4.0 ± 2.7 mM (n = 3), which was nearly consistent with the Michaelis constant (Km) values obtained by the electrophysiological analysis (Km = 0.5–1.8 mM) (18, 19, 20), transport analysis using RI-D-glucose (Km = 0.51 mM) (21), and intermolecular interaction analysis with the purified SGLT1 protein (Km = 0.84 mM) (34).

**Figure 4:**
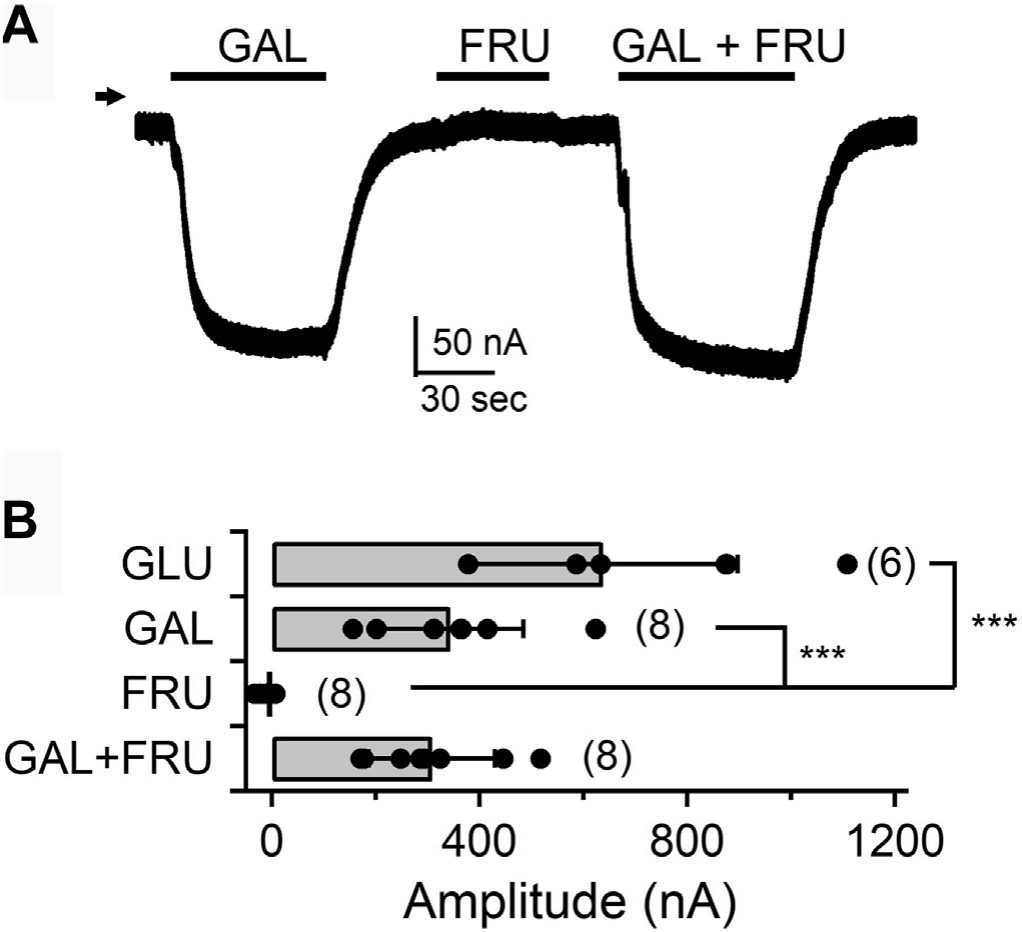
**Effects of D-fructose on the sugar transport of hSGLT1.***A*, representative current trace of hSGLT1 WT by application of 10 mM D-galactose and D-fructose and both D-galactose and D-fructose. *B*, comparison of current amplitudes induced by 10 mM sugar. Bars indicate the means ± SD, and all data are plotted as *black dots*. Numbers in brackets indicate the number of experiments (n = 6–8). Data were statistically analyzed *via* one-way ANOVA with Tukey–Kramer’s post hoc test (∗∗∗*p* < 0.001). FRU, D-fructose; GAL, D-galactose; SGLT, sodium–glucose cotransporter.

**Figure 5:**
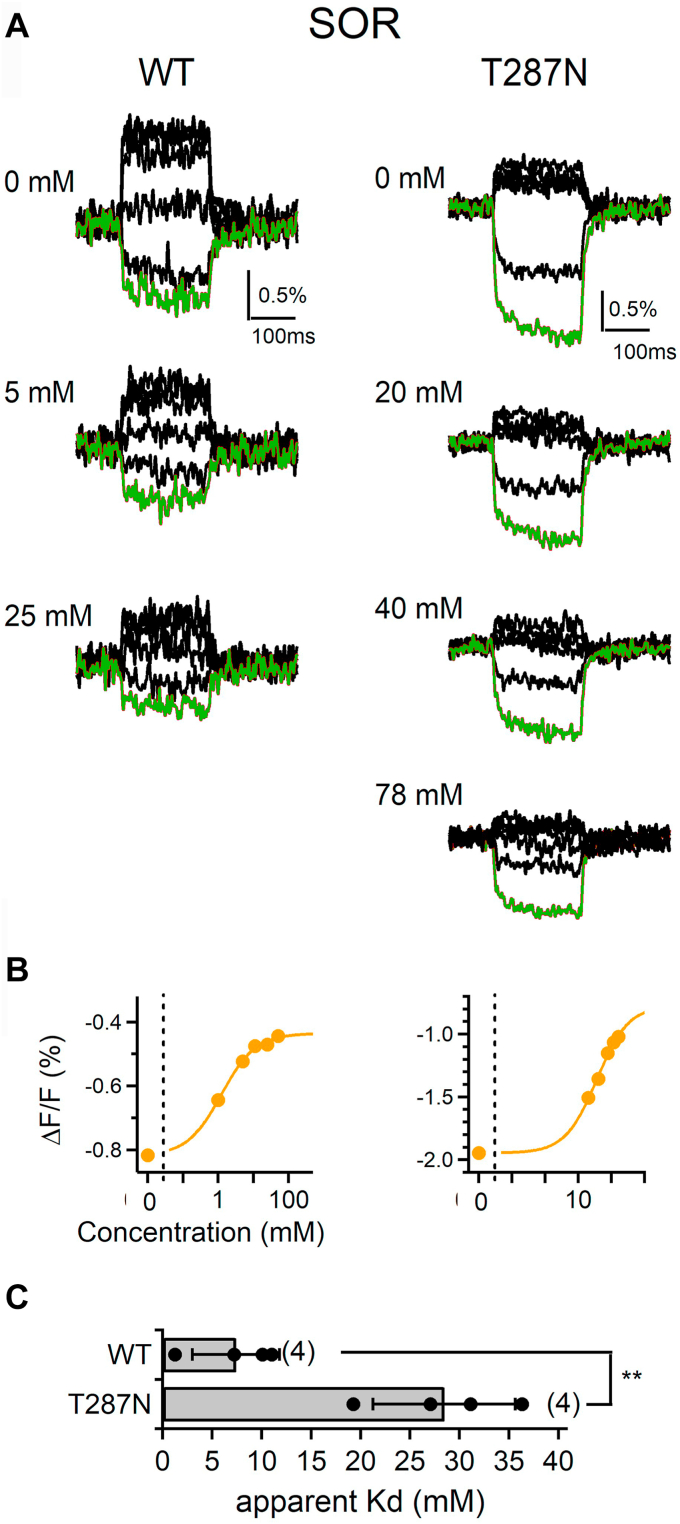
**Effects of L-sorbose on the voltage-induced fluorescence changes of hSGLT1 WT and T287N.***A*, representative voltage-induced fluorescent traces of hSGLT1 measured with different concentrations of L-sorbose. Step pulses were applied as in Figure 1*D*. *Red traces* show each fluorescent signal obtained by the −200 mV step pulse. *B*, dose–response relationships of the fluorescent intensity of hSGLT1 WT and T287N. Fluorescent values at the end point of test pulse at the −200 mV step in A were plotted for 1, 5, 11, 25, and 50 mM in WT and for 20, 40, 78, 116, and 165 mM in T287N (*orange circle*), and Hill-fitted curves are also shown. *C*, comparison of the apparent Kd values. Bars indicate the means ± SD, and all data are plotted as *black dots* (n = 3–4). Data were statistically analyzed with student’s *t* test (∗∗*p* = 0.0025 < 0.01). SGLT, sodium–glucose cotransporter; SOR, L-sorbose.

We then performed binding experiments on other monosaccharides using the same hSGLT1 G507C-TAMRA construct used for the VCF analysis. Representative fluorescence signal traces before and after monosaccharide administration are shown (Fig. 2*A*). The dose-*ΔF/F* relationship between the concentration of sugar and the change in fluorescence measured when −200 mV was applied is shown in Figure 2*B*. The obtained apparent Kd values are shown in Figure 2*C*. The VCF analysis showed that hSGLT1 also binds to D-galactose, which is consistent with the transport capacity of hSGLT1. A concentration-dependent decrease in VCF signaling was also observed following the administration of D-fructose, a substrate not transported by hSGLT1, suggesting that D-fructose binds to hSGLT1. The apparent Kd values of D-galactose and D-fructose were 10.2 ± 4.2 mM (n = 5) and 5.1 ± 2.9 mM (n = 4), respectively (Fig. 2*C*). The apparent Kd value of D-galactose was nearly consistent with the Km values obtained by electrophysiological analysis (Km = 0.6–6.1 mM) (18, 19, 20), transport analysis using RI-D-galactose (Km = 0.56 mM) (21), and intermolecular interaction analysis with the purified SGLT1 protein (Km = 0.86 mM) (34). The apparent Kd value for non-transported D-fructose was not significantly different from that for D-glucose. hSGLT1 WT has been reported to have very low transport activity for D-allose (ALL) compared with D-glucose (20). When D-allose was applied in the VCF analysis, the decrease in the fluorescent signal was low and not observed until higher concentrations were administered (Fig. 2, *A* and *B*); therefore, the apparent Kd values of D-allose could not be calculated (Fig. 2*C*). This is consistent with the Km value obtained by electrophysiological analysis (Km > 100 mM) (20). We also performed a VCF analysis using D-ribose (C_5_H_10_O_5_), a monosaccharide that does not have the C_6_H_12_O_6_ structure analyzed above and is not transported by SGLT1; therefore, no analyzable affinity was obtained (Fig. 2*A*).

Next, we performed binding experiments using the hSGLT1 T287N/G507C-TAMRA construct for VCF analysis. The T287N mutant substituted for the sequence of sodium/galactose symporter of *Vibrio parahaemolyticus* has been reported to cause steric hindrance to D-glucose in the binding pocket, thereby preserving D-galactose transport but significantly reducing D-glucose transport (20). Representative fluorescent signal traces before and after monosaccharide administration are shown (Fig. 3*A*). The dose-*ΔF/F* relationship measured when −200 mV was applied is shown (Fig. 3*B*), and the apparent Kd values are shown (Fig. 3*C*). As expected, the T287N mutation had a higher binding affinity for D-galactose than for D-glucose, with values of 230 ± 200 mM (n = 5) for D-glucose and 68 ± 31 mM (n = 4) for D-galactose (Fig. 3*C*). Moreover, a marked decrease in D-glucose affinity observed for the T287N mutation, which is consistent with previous reports using electrophysiology (20). This result suggests that the binding affinities of D-glucose and D-galactose to SGLT obtained from the VCF analysis reflect the binding of substrates to the binding pocket. In addition, a concentration-dependent decrease in the fluorescence signal by application of D-fructose was observed for T287N, indicating the binding of D-fructose, and the apparent Kd value was 29 ± 9 mM (n = 6) (Fig. 3*C*).

Although previous reports indicated that D-fructose is non-transported, this study found that it binds to hSGLT1. We hypothesized that the binding of D-fructose to hSGLT1 affects its substrate transport function. We analyzed the monosaccharide transport function using electrophysiological analysis. Representative Na^+^ current traces were measured using two-electrode voltage-clamp recordings when different sugars were applied to the hSGLT1 WT expressed in oocytes (Fig. 4*A*). The measured current amplitudes are shown as bar graphs (Fig. 4*B*). Na^+^ transport currents induced by D-glucose and D-galactose were not induced by D-fructose (Fig. 4, *A* and *B*). The amplitude of the current induced by D-galactose was unchanged upon the addition of D-fructose (Fig. 4, *A* and *B*). These results indicate that the binding of D-fructose observed in the VCF experiments did not affect the transport function of hSGLT1.

Substrates of hSGLT1, such as D-glucose and D-galactose, exhibit a pyranose structure, whereas D-fructose has a furanose structure (13). However, D-fructose is known to be in equilibrium between the furanose and pyranose states in aqueous solution, with 71% D-fructopyranose, 28% D-fructofuranose, and 1% ketofructose (35). L-sorbose is a ketohexose-like D-fructose that exhibits the pyranose structure (L-sorbopyranose) at a proportion of more than 98% in aqueous solution (36, 37). L-sorbopyranose differs from D-fructopyranose only in the orientation of one OH group. We performed binding experiments with L-sorbose using VCF analysis, and the representative fluorescence signal traces (Fig. 5*A*), dose-*ΔF/F* relationship (Fig. 5*B*), and apparent Kd values were determined (Fig. 5*C*). The hSGLT1 WT- and T287N-based TAMRA constructs both exhibited a binding affinity to L-sorbose as high as that to D-fructose. The apparent Kd values were 7.4 ± 4.4 mM (n = 4) for the WT and 28.4 ± 7.2 mM (n = 4) for T287N (Fig. 5*C*). Based on these results, it is likely that fructose binds to hSGLT1 in a pyranose state.

## Discussion

In this study, substrate specificity of SGLT was determined by performing a VCF analysis of the binding affinity of monosaccharides to hSGLT1 expressed in *Xenopus* oocytes. The results showed that the hSGLT1 WT bound with high affinity to the transported substrates D-glucose and D-galactose. Furthermore, the binding of non-transported fructose was observed (Fig. 2). In the T287N mutant, which exhibited attenuated D-glucose transport relative to D-galactose, the binding affinity of D-galactose exceeded that of D-glucose, and D-fructose was also bound (Fig. 3). Electrophysiological analysis revealed that D-galactose-induced Na^+^ currents were not inhibited by D-fructose (Fig. 4). The binding of L-sorbose, mostly in the L-sorbopyranose form in solution, was also observed for both the SGLT1 WT and T287N mutant (Fig. 5).

Transporters bind to the substrate from one side of the plasma membrane, cause a conformational change, and release and transport the substrate to the other side of the plasma membrane. In SGLT, Na^+^ and sugar bind to their respective binding pockets and are cotransported according to the Na^+^ concentration gradient. The binding affinity of the sugar was estimated from its transport activity in terms of enzyme reaction kinetics using electrophysiological analysis and transport assays with RI-labeled substrates (16, 18, 19, 20, 34). In this study, the binding affinity of sugars were determined based on the VCF analysis according to the weakening of the fluorescent signal after applying a transport substrate as an indicator. The apparent Kd values were almost within the same range as the Km values estimated from previous electrophysiology and transport analyses in hSGLT1 WT and T287N (16, 18, 19, 20, 34), thus supporting the usefulness of this methodology. Furthermore, this VCF assay can analyze the binding affinity between SGLT and sugars on the living cell membrane and provides more physiological information than molecular interaction analyses using purified proteins. In fact, the apparent Kd values for D-glucose determined in this study are comparable to those of human blood glucose at 5.5 to 7.7 mM (38), which may be the appropriate affinity for binding and unbinding *in vivo*.

The mechanism of sugar selectivity has been studied by functional analysis, structural biological analysis, mutation experiments, and molecular dynamics simulations; however, analyses of the binding pocket structure have not provided a complete explanation. Therefore, studies have proposed that substrate selection is acquired by the presence of binding sites other than the known binding pocket or by changes in the structure of the binding pocket during transport (18, 20, 25). In the present study, the binding of D-fructose, a nontransported substrate, to the hSGLT1 WT supports these findings. One possibility is that D-fructose binds to a site that is positionally and functionally independent of the binding pocket and alters the fluorescence signal as indicated by the VCF analysis. However, this is unlikely because the experimental results with T287N suggested that the change in the fluorescence signal was due to substrate binding to the binding pocket (Fig. 3). Compared to the kinetics of the hSGLT WT, faster VCF kinetics were observed for T287N, which has a lower sugar binding affinity (Figs. 2*A* and 3A). Slower kinetics were also observed in T287N treated with D-galactose, which has relatively preserved affinity (Fig. 2*A*). Kinetics may reflect voltage-induced conformational changes in SGLT, and differences in kinetics may be more clearly observed in T287N when the transported sugar is bound or unbound. Although the present study focused on sugar binding, future studies investigating the conformational changes in sugar transport using VCF signal analysis will provide a more advanced understanding of transport function.

In our previous study, a quadruple mutant of hSGLT1 was generated to match the sequence of hSGLT4 that transports D-fructose and fructose and the structurally similar L-sorbose and D-mannose (20). Structural modeling analysis suggested that D-fructose fit the binding pocket of the hSGLT1 quadruple mutant in a pyranose structure (20). These reports and the binding of D-fructose and L-sorbose to hSGLT1 reported here suggest that D-fructopyranose and monosaccharides of similar structures can bind to the hSGLT1 binding pocket, regardless of their transport. The finding that the nontransported substrates D-fructose and L-sorbose bind to hSGLT1 is valuable. Although our results indicate that they did not inhibit substrate transport, it may be possible to create compounds that inhibit transporter activity by adding a modified group to D-fructose or L-sorbose. Most SGLT2 inhibitors currently available on the market are derived from the structure of glucose (39). The development of new drugs with fewer side effects based on nonglucose sugars with low biological reactivity is promising.

## Experimental procedures

### Molecular biology

This molecular biology analysis method has been previously described (20). The EcoRI-XbaI fragment of human SGLT1 (NCBI Reference Sequence: NW_00343.4; a gift from Dr T. Makino) was subcloned into the pGEMHE vector (40), and each point mutation (G507C and T287N) was introduced using the PrimeSTAR Mutagenesis Basal Kit (TAKARA BIO) and verified by DNA sequencing. Complementary RNAs (cRNAs) were produced by transcription of linearized plasmids using the mMESSAGE mMACHINE T7 Transcription Kit (Thermo Fisher Scientific Inc).

### Preparation of *Xenopus* oocytes

All experiments were performed in accordance with the guidelines of the Animal Research Committee of Kagawa University, Japan, and the basic guidelines for animal experiments in the field of physiology of the Physiological Society of Japan.

Oocytes were collected from an adult female *Xenopus laevis* anesthetized in ice-cold pure water supplemented with 0.2% ethyl methanesulfonate and 3-aminobenzoate (Sigma-Aldrich) for 30 to 60 min, as described previously (20). The isolated oocytes were then treated with type I collagenase (1.0 mg/ml, Sigma-Aldrich) for 2 to 3 h, injected with approximately 50 nl of cRNA, and incubated for 2 to 3 days at 18 °C in modified Barth’s Solution [MBSH: 88 mM NaCl, 1 mM KCl, 2.4 mM NaHCO_3_, 0.3 mM Ca(NO3)2, 0.41 mM CaCl_2_, 0.82 mM MgSO_4_, and 15 mM Hepes (pH 7.6)].

### Voltage-clamp fluorometry

Oocytes were injected with 50 nl of hSGLT1 G507C or hSGLT1 G507C/T287N cRNA (0.1–0.2 ng/nl) per oocyte and incubated in MBSH buffer for 2 to 3 days at 18 °C. hSGLT1 G507C expressed in the oocytes was labeled by incubating with MTS-TAMRA (10 μM) (Toronto Research Chemicals) for 60 min at 18 °C in the dark, and then the oocytes were washed twice in MBSH. Subsequently, the oocytes were imaged using a macrozoom fluorescence microscope (MVX10; Olympus) equipped with an objective lens (MVPLAPO 2 XC; Olympus) and a high-intensity LED lamp (Thorlabs Inc) under a two-electrode voltage clamp to control the oocyte membrane potential. BP535-555, BP570-625, and DM565 (Olympus) were used as the excitation filter, emission filter, and dichroic mirror, respectively. Fluorescence was detected using a photomultiplier tube (H10722–20; Hamamatsu Photonics). The photomultiplier tube output was digitized using an AD/DA converter (Digidata 1550 B; Molecular Devices) with a two-electrode voltage clamp setup (41). Glass microelectrodes filled with 3 M KCl were used as intracellular electrodes. Their electrical resistances ranged from 0.5 to 1.0 Mohm, and recordings were performed at room temperature in a bath solution [90 mM NaCl, 3 mM KCl, 0.74 mM CaCl_2_, 0.82 mM MgCl_2_, and 10 mM Hepes (pH 7.5)]. As a typical voltage protocol, the membrane potential was held at −60 mV, and step pulses of 200 ms were applied in 40 mV increments from −200 to 200 mV, with the DC gain booster of the amplifier turned on. The sugars added to the bath solution were D-glucose, D-fructose, D-ribose (FUJIFILM Wako Chemicals), D-galactose (Sigma Aldrich), D-allose (Matsutani Chemical Industry), and L-sorbose (Nacalai Tesque Inc). The averaged fluorescent traces from eight traces were digitally filtered at 100 to 300 Hz.

### Two-electrode voltage-clamp

Oocytes were injected with 50 nl of hSGLT1 WT cRNA (0.1–0.2 ng/nl) per oocyte and incubated in MBSH buffer at 18 °C for 2 to 3 days. Macroscopic currents from *Xenopus* oocytes were measured by ligand-substrate administration using established protocols (42, 43, 44) and recorded from oocytes expressing the hSGLT1 WT using a bath-clamp amplifier (OC-725D, Warner Co), an AD/DA converter, and pClamp 11 recording software (Molecular Devices). Glass microelectrodes filled with 2 M CH_3_COOK and 1 M KCl (pH 7.2) were used as intracellular electrodes, and their electrical resistances ranged from 0.2 to 0.4 Mohm. Induced Na^+^ currents by sugar administration were recorded at a holding potential of −60 mV by turning on the DC gain booster of the amplifier in the bath solution at room temperature. The recordings were performed under continuous perfusion using a VC3-8PG perfusion system (ALA Scientific Instruments, Inc), with sugars administered in the bath solution (20).

### Data analysis

In all experiments, data were analyzed as biological replicates. The same recording protocol was applied to oocytes expressing hSGLT1. The number of samples (n) is indicated in the figures or figure legends. Experimental data are shown as the means ± standard deviation. In the VCF analysis, the normalized fluorescence intensity (*ΔF/F*) was obtained by dividing the change in fluorescence at −200 mV hyperpolarization (*ΔF*) by the baseline fluorescence intensity at the holding potential (*F*). As the expression of transporters and amount of attached fluorescent reagent varied from oocyte to oocyte, the VCF signal amplitude also varied. Therefore, we measured the VCF signal from the same oocyte in a series with different sugar concentrations to analyze the affinity. The mean of the response amplitudes for each sugar concentration was plotted and fitted using the Hill equation to determine the apparent Kd values. The Levenberg–Marquardt algorithm implemented in Igor Pro (WaveMetrics, Inc) was used to compute initial estimates for the curve fitting. In the fitting, parameters were not fixed, and fitting was performed within the range of points of the experimental data. The apparent Kd value obtained by Hill fitting was considered as the apparent sugar affinity. Data were statistically analyzed *via* Student's *t* test or one-way ANOVA with Tukey–Kramer’s post hoc test. The data were analyzed using Clampfit (Molecular Devices), Igor Pro, and SPSS (IBM).

## Data availability

All data are contained within the manuscript.

## Conflict of interest

The authors declare that they have no conflicts of interest with the contents of this article.
